# Association of Atraumatic Splenic Rupture and Acute Pancreatitis: Case Report with Literature Review

**DOI:** 10.1155/2022/8743118

**Published:** 2022-02-14

**Authors:** Lidija Ljubicic, Vibor Sesa, Silvija Cukovic-Cavka, Ivan Romic, Igor Petrovic

**Affiliations:** ^1^Department for Respiratory Diseases Jordanovac, University Hospital Centre Zagreb, Zagreb, Croatia; ^2^Department of Gastroenterology, University Hospital Centre Zagreb, Zagreb, Croatia; ^3^School of Medicine, University of Zagreb, Zagreb, Croatia; ^4^Department of Surgery, University Hospital Centre Zagreb, Zagreb, Croatia

## Abstract

Atraumatic splenic rupture is an uncommon complication of acute pancreatitis. This article presents a case of a 35-year-old patient presenting with acute pancreatitis who subsequently developed a splenic vein thrombosis and splenic rupture requiring a laparotomy and splenectomy. This rare but life-threatening complication requires prompt recognition and management in patients with pancreatitis who develop sudden hemodynamic instability.

## 1. Introduction

Atraumatic splenic rupture (ASR) is a rare clinical disorder that can develop in a diseased spleen. A literature review by Renzulli et al. found neoplasia (30.3%) as the most frequently reported pathology in ASR followed by infectious (27.3%), inflammatory, and non-infectious (20.0%) causes [1]. When it comes to infections, malaria represents the single major cause of ASR worldwide [2]. Splenic rupture is also an infrequent but life-threatening complication of severe Babesia microti infection. Babesiosis is transmitted mainly through bites from infected Ixodes scapularis ticks, and the incidence of tick-borne diseases in the US is increasing. In a systematic review published in 2020, Dumic et al. described the clinical features, laboratory findings, and the management of patients with splenic complications during the acute infection with Babesia microti [3]. Spontaneous rupture of the spleen, secondary to infectious mononucleosis (caused by the Epstein-Barr virus), is also rare. Still, it is the most frequent cause of death in infectious mononucleosis [4]. On the contrary, the term “idiopathic” ASR suggests rupture of normal-appearing spleen without predisposing factors [2]. The anatomical proximity of the pancreatic tail and the splenic hilum makes the spleen susceptible to inflammatory processes involving the pancreas. The pathophysiological mechanisms leading to spleen lesions in acute pancreatitis are not fully known. However, several theories have been proposed, such as direct spleen erosion caused by pancreatic pseudocysts, pancreatic enzyme extravasation, perisplenic adhesions due to recurrent inflammation of the pancreas, and spleen congestion following splenic vein thrombosis [5]. Splenic rupture is more often described as a complication of chronic pancreatitis, but the number of ruptures in the setting of acute pancreatitis is growing. Other possible splenic complications of pancreatitis include arterial pseudoaneurysm, perisplenic/intrasplenic pancreatic pseudocyst, infarction, splenic subcapsular infection, hematoma, and necrosis [6]. According to the available literature, the exact incidence and epidemiology of these events are not known, but as stated in one systematic review, about 10% of atraumatic ruptures of the spleen are associated with inflammatory processes of the pancreas [1]. We report a case of a middle-aged patient with acute pancreatitis and splenic vein thrombosis complicated by splenic rupture.

### 1.1. Ethical Consideration

There is no ethical approval at our Institution for case reports.

## 2. Case Report

**Figure 1:**
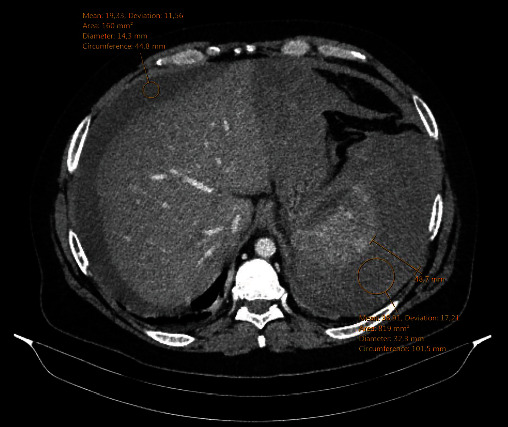
Perisplenic hematoma up to 5 cm wide with intraperitoneal haemorrhagic content in perihepatic and gastrohepatic space.

A 35-year-old man was admitted to the emergency department and presented with symptoms typical for acute pancreatitis: pain in the upper abdomen radiating to both rib arches accompanied by vomiting of four hours. The patient reported occasional alcoholic abuse, including alcohol and fatty meal consumption, before the onset of symptoms. He did not use any drug regularly and had no positive medical history of any specific disease. During the initial physical examination, the patient was hemodynamically and respiratory stable (150/90 mmHg, pulse 84/min, and spO2 98%). There were no pathologic findings except mild epigastric tenderness without rebound or guarding. Laboratory exams showed elevated haemoglobin (178 g/L), leukocytosis with neutrophilia (27.2 × 10^9^/L, 79% neutrophils), hyperglycaemia (12.3 mmol/L), high levels of pancreatic enzymes (amylase 3156 U/L and lipase 5819 U/L), and a mild liver lesion (bilirubin 40 *μ*mol/L, alkaline phosphatase 109 U/L, gamma-glutamyl transferase 300 U/L, and alanine aminotransferase 68 U/L). Despite significant inflammatory response in pancreatitis, the initial serum level of C-reactive protein was normal. Abdominal ultrasound described cholecystolithiasis without cholecystitis. The patient was hospitalised and treated conservatively with crystalloid infusions, restriction of oral intake, and analgetic therapy. During the next day, additional laboratory evaluation was performed, which showed the normalisation of the haemoglobin value (163 g/L), a decrease in leukocytes (L 16.6 × 10^9^/L), and serum calcium value (1.92 mmol/L) with normal cholesterol and triglyceride level, but an increase in CRP (69.6 mg/L) and glycaemia (23 mmol/L). On the third day, a clinical deterioration was noted as the patient complained of light-headedness, sweating, and generalised malaise. He was hypotensive at a blood pressure of 90/70 mmHg and tachycardic at a heart rate of 150 beats/min. Due to clinical instability, he was transferred to the intensive care unit (ICU). The abdominal ultrasonography described free fluid in the peritoneal cavity and suspected splenic hematoma. As shown in Figure 1, the abdominal computed tomography (CT) revealed a splenic rupture with perisplenic hematoma up to 5 cm wide, intraperitoneal haemorrhagic content, edematous parenchyma of the pancreas with peripancreatic collections, and gallbladder stones. Partial portal vein thrombosis and superior mesenteric vein with complete splenic vein thrombosis have also been described.

The patient underwent an urgent laparotomy, which showed extensive hemoperitoneum due to the rupture of the splenic parenchyma, splenic vein thrombosis, and pancreas necrosis with only a small area of vital tissue along with the descending duodenum. Evacuation of the hematoma, splenectomy, distal pancreatectomy, and cholecystectomy were performed. A drain was placed in the area of the head of the pancreas. Histopathological examination of tissues revealed splenic parenchyma impregnated with haemorrhagic contents and almost complete necrosis of the resected pancreatic parenchyma. The patient was postoperatively transferred to an ICU. He was treated with fluid resuscitation, blood transfusions, and antibiotic administration. Parenteral nutrition was initiated, and a pneumococcal vaccine was given. Due to the persistently high values of CRP, abdominal distension, and CT showing dilated small bowel loops with the progression of the pancreatic head necrosis, reoperation was performed on the 8th postoperative day, and it revealed dilated small bowel loops and necrotic collection in the area of the pancreatic head. A complete adhesiolysis was done with necrosectomy in the pancreatic head area with a sump drain left in place. Postoperative monitoring of drain fluid amylase (DFA) levels revealed levels >16 000 U/L, which, in addition to MSCT findings, suggested the existence of postoperative pancreatic fistula (POPF), and we treated the patient with octreotide, administered subcutaneously in a dose of 200 *μ*g/day.

A chest drainage tube was also inserted due to pleural effusion. Postoperatively, a significant decrease in inflammatory markers was noted, and orderly, peristalsis was established as well as the oral intake. The wound healed by primary intention.

A control computed tomography scan of the abdomen showed resolution of the thrombi within the lumen of the portal vein. Due to the development of secondary diabetes, an endocrinologist was consulted, and insulin therapy started. The further postoperative course was uneventful, and the patient was discharged in stable condition.

Nineteen days after being discharged, he required readmission due to abdominal pain and fever that started two days earlier. Laboratory exams showed leukocytosis (13.8 × 10^9^/L) with C-reactive protein 151.8 mg/L and elevated amylase levels in urine. MSCT revealed multilocular collection characteristics of abscesses that were indistinctly separable from the duodenum's distal segment and required CT-guided percutaneous drainage. The drained fluid was infected with Pseudomonas aeruginosa isolated, and antibiotic therapy with colistin was initiated. The patient was discharged with a drain left in place for the next six months when the complete cessation of secretion was noted, and the drain was removed. At one-year follow-up, the patient is free of symptoms.

## 3. Literature Review

A literature search of the PubMed/MEDLINE/Google Scholar (January 2010 to April 2021) databases was conducted. We found 22 published articles dealing with the subject.  Table 1 summarises cases found in the literature. The mean age was 44.0 years, and most were men (71%). Out of 24 cases, 20 patients (80%) presented with acute pancreatitis and remain presented with chronic pancreatitis. Alcohol was the aetiology in 17 (71%) of cases. Other causes included corticosteroid therapy, hyperlipidemia, and Crohn's disease (CD), although the latter is not entirely clarified because the patient had CD involving only the distal colon. In the remaining 4 cases, the cause of pancreatitis was unknown. All the patients suffered from more or less abdominal pain, and four patients had Kehr's sign (acute pain at the tip of the shoulder due to the presence of blood or other irritants in the peritoneal cavity). More than half of the rupture cases (84%) were managed by laparotomy with or without splenectomy. Eight cases were treated conservatively. Splenic vein thrombosis was noted in seven patients. Left pleural effusion was described in three patients. Effusions related to structures lying beneath the hemidiaphragm are less frequently seen but well documented. However, its frequency is not well known in the absence of data.

## 4. Discussion

Spontaneous splenic haemorrhage is rare yet an important complication of pancreatitis. The spleen is a highly vascularised organ that can, in the case of haemorrhage, result in significant blood loss either from the parenchyma or the arteries and veins that supply the spleen. The exact incidence and epidemiology have not been clearly defined in the literature, but splenic complications in chronic pancreatitis tend to favour men [7]. In most cases, alcohol use, gallstones, and hypertriglyceridemia cause acute pancreatitis. It can also be drug-induced or may occur postprocedural. The rate of occurrence of each aetiology of acute pancreatitis varies across geographic regions. In a literature review published in 2020, Jain et al. described 28 cases of pancreatitis with splenic complications. The majority of the patients were men, and the most common aetiology was alcohol consumption, as in our case.

Complications of chronic pancreatitis mainly include the development of subcapsular hematoma, splenic pseudocyst, and rupture of the spleen and in acute pancreatitis splenic infarction and bleeding within the spleen parenchyma [8]. The average onset of these events is two years from diagnosis [9]. The leading cause of these complications is the close anatomical relationship between the tail of the pancreas and the hilus of the spleen. The splenic artery and vein together with the tail of the pancreas enter the hilus of the spleen. Direct communication facilitates the development of splenic hematomas, abscesses, and pseudocysts. Direct extravasation of pancreatic enzymes, splenic vein thrombosis, and consequent splenic congestion are some of the proposed pathophysiological mechanisms leading to these complications [5, 6]. Unfortunately, in most of the presented cases, the initial prognostic factors of pancreatitis severity were not determined as a possible additional risk factor for the development of splenic complications. The clinical presentation is often nonspecific except for the spleen rupture that most often manifests itself in the form of progressive pain below the left costal arch with or without radiation to the left shoulder (Kehr's sign), fever, and palpable mass under the left costal arch on physical examination. The common symptoms in this study were abdominal pain in the left hypochondrium associated with nausea and vomiting. Our patient presented with signs of hemorrhagic shock. He also had a left pleural effusion, a significant indicator of splenic complications [10].

The MSCT scan is the diagnostic modality of choice in patients with suspected splenic complications. It may show disruption in the normal splenic parenchyma, surrounding hematoma, intraparenchymal hematoma, and free intra-abdominal blood and also evaluates and categorises splenic injuries according to the American Association for the Surgery of Trauma (AAST) scale [2]. A contrast-enhanced CT scan should be obtained to determine the density difference between the splenic parenchyma and hematoma. Although splenectomy was the traditional management of spontaneous splenic rupture, numerous recent reports have documented positive outcomes with nonoperative management, so this treatment modality is increasingly evolving. Nonoperative management can be successful in hemodynamically stable patients, and in most cases reported, arterial embolisation and percutaneous drainage were effective for treatment. Hasegawa et al. recently reported a case in which they used EUS-guided drainage to successfully treat splenic rupture caused by a pancreatic pseudocyst. The most important precondition for successful selective nonoperative management is adequate patient selection.

## 5. Conclusion

Atraumatic splenic rupture is a rare complication of pancreatitis. Previously, it was more often described as a complication of chronic pancreatitis. However, the number of described ruptures in the setting of acute pancreatitis has been growing, and our data supported this finding. A computed tomography scan is the diagnostic modality of choice. Treatment predominantly depends on the patient's hemodynamic stability and clinical assessment. The hemodynamically unstable patient with splenic rupture or haemoperitoneum will require emergency laparotomy and splenectomy or distal pancreatosplenectomy. In hemodynamically stable patients, splenic artery embolisation, percutaneous collection drainage, and conservative approach can be considered. It is important to emphasise the importance of an early suspicion of atraumatic splenic rupture as a complication of pancreatitis because of its rarity and fatal consequences if not recognised on time.

## Figures and Tables

**Table 1:** Summary of reported cases.

Age/gender	Type of pancreatitis	Etiology	SVT^∗^	Splenic rupture	Treatment	Outcome	Study	Year
35/M	Acute	Unknown	No	Yes	Surgical	Recovery	Gandhi et al.	2010 [11]
37/M	Acute	Crohn's disease	Yes	Yes	Conservative	Recovery	Mujtaba et al.	2011 [12]
45/M	Acutisation	Alcohol	No	Yes	Surgical	Recovery	Jha et al.	2011 [13]
47/M	Acutisation	Alcohol	No	Yes	Surgical	Recovery	Patil et al.	2011 [14]
23/F	Acute	Alcohol	Yes	Yes	Surgical	Recovery	Patil et al.	2011 [14]
45/F	Acute	Idiopathic	No	No	Conservative	Recovery	Patil et al.	2011 [14]
47/F	Acute	Alcohol	No	No	Conservative	Recovery after drainage	Lee et al.	2012 [15]
38/F	Acute	Alcohol	No	Yes	Surgical	Recovery	Cengiz et el.	2013 [16]
55/M	Chronic	Unknown	Yes	No	Conservative	In recovery	Sawrey et al.	2013 [17]
45/M	Acute	Alcohol	No	Yes	Surgical	Death	Debnath et al.	2014 [18]
65/F	Acute	Hypertriglyceridemia	Yes	No	Conservative	Recovery	Gündüz et al.	2015 [19]
25/M	Chronic	Alcohol	No	Yes	Surgical	Recovery	Sharada et al.	2015 [20]
30/M	Acute	Alcohol	Yes	Yes	Surgical	Recovery after drainage	Hernani et al.	2015 [21]
29/M	Chronic	Alcohol	No	Yes	Surgical	Recovery	Moori et al.	2016 [22]
59/M	Acute	Alcohol	Yes	Yes	Surgical	Recovery	Zhou et al.	2016 [23]
48/M	Acutisation	Alcohol	No	Yes	Conservative	Recovery	Sanchez et al.	2017 [24]
63/M	Acute	Alcohol	No	Yes	Surgical	Recovery	Fenando et al.	2019 [25]
60/M	Acutisation	Alcohol	No	Yes	Surgical	Recovery	Balanis et al.	2019 [26]
39/M	Acute	Alcohol	No	Yes	Conservative	Recovery	Zarrin et al.	2019 [27]
49/F	Acutisation	Idiopathic	Yes	Yes	Surgical	Recovery	Jain et al.	2019 [5]
40/M	Acute	Alcohol	No	No	Surgical	Recovery	Mukherjee et al.	2020 [28]
43/M	Chronic	Alcohol	No	Yes	Conservative	Recovery	Hasegawa et al.	2020 [29]
26/M	Acute	Alcohol	No	Yes	Surgical	Recovery	Navarro et al.	2021 [30]
63/F	Acute	Steroid-induced	No	Yes	Surgical	Recovery	Nadaraja et al.	2021 [8]

^∗^SVT: splenic vein thrombosis.

## Data Availability

Clinical data were obtained by reviewing medical records.

## Abbreviations

CRP: C-reactive protein
ICU: Intensive care unit
CT: Computed tomography
DFA: Drain fluid amylase
POPF: Postoperative pancreatic fistula
CD: Crohn's disease
AAST: American Association for the Surgery of Trauma.
